# Silver Nanowire/Colorless-Polyimide Composite Electrode: Application in Flexible and Transparent Resistive Switching Memory

**DOI:** 10.1038/s41598-017-03746-1

**Published:** 2017-06-13

**Authors:** Seung-Won Yeom, Banseok You, Karam Cho, Hyun Young Jung, Junsu Park, Changhwan Shin, Byeong-Kwon Ju, Jong-Woong Kim

**Affiliations:** 1 Display and Nanosystem Laboratory, College of Engineering, Korea University, Anam-dong, Seoul 139-713 Republic of Korea; 2 Display Materials & Components Research Center, Korea Electronics Technology Institute, Seongnam, 463-816 Republic of Korea; 3 School of Electrical and Computer Engineering, University of Seoul, 163 Seoulsiripdae-ro, Dongdaemun-gu, Seoul 130-743 Republic of Korea; 4 Department of Energy Engineering, Gyeongnam National University of Science and Technology, Jinju, 52725 Republic of Korea

## Abstract

Improving the performance of resistive switching memories, while providing high transparency and excellent mechanical stability, has been of great interest because of the emerging need for electronic wearable devices. However, it remains a great challenge to fabricate fully flexible and transparent resistive switching memories because not enough research on flexible and transparent electrodes, for their application in resistive switching memories, has been conducted. Therefore, it has not been possible to obtain a nonvolatile memory with commercial applications. Recently, an electrode composed of a networked structure of Ag nanowires (AgNWs) embedded in a polymer, such as colorless polyimide (cPI), has been attracting increasing attention because of its high electrical, optical, and mechanical stability. However, for an intended use as a transparent electrode and substrate for resistive switching memories, it still has the crucial disadvantage of having a limited surface coverage of conductive pathways. Here, we introduce a novel approach to obtain a AgNWs/cPI composite electrode with a high figure-of-merit, mechanical stability, surface smoothness, and abundant surface coverage of conductive networks. By employing the fabricated electrodes, a flexible and transparent resistive memory could be successfully fabricated.

## Introduction

The confrontation between current conventional silicon-based memory technologies and the limits of miniaturization has given rise to the development of emerging memory technologies such as ferroelectric random access memories (FRAMs), magnetic RAMs (MRAMs), phase-change RAMs (PRAMs), and resistive RAMs (RRAMs)^1–4^. These emerging memories store information based on the bistability of materials by taking advantage of changes in their physical properties. Among these memories, RRAMs are considered one of the best candidates for the development of next-generation nonvolatile memory devices due to their fast switching speed, low energy consumption, excellent endurance, long retention, and simple metal-insulator-metal structure^1, 2^.

Resistive switching in RRAMs can be achieved through the formation and rupture of a conductive filament formed in their insulation layer, resulting in low and high resistance states. The switching process is caused by the motion of charged ions, which can be driven by an applied electric field and the ionic species present. Based on the polarities of the charges, two mechanisms are suggested to explain the ionic motion: the first one is through cation migration, which is found in some chalcogenides, oxides, amorphous Si, and organic materials^1, 3^; and the other is through anion migration (e.g. oxygen vacancies)^1, 4^. A cation migration-based RRAM is usually fabricated using an electrochemically active electrode such as Ag or Cu, and an electrochemically inert counter electrode such as Pt, Au, or W. Cation migration-based RRAMs have several advantages such as fast switching, low power consumption, high scalability, and superior switching endurance when compared with anion migration-based RRAMs^5^. In general, solid electrolytes have been used in cation-based RRAMs due to their high cation mobility.

Recently, the memory with low power consumption, low cost and fast speed is no longer the only standard for emerging technological needs. The memory with sophisticated functionalities such as flexibility and transparent has tremendous advantages and additional value in different areas for the future electronics applications. In particular, for fully integrated electronic systems, the functional memory unit is one of the most essential and fundamental components for information storage and process in numerous products. In order to fabricate highly transparent RRAM devices, wide band-gap resistance change materials sandwiched between transparent conductive electrodes are necessary^6–9^. In this respect, TiO_2_-based RRAMs have attracted attention because of their excellent electrical performance, transparent characteristics in the visible-light spectrum, as well as superior mechanical flexibility^10, 11^. These properties have prompted their use in flexible and transparent RRAMs (FT-RRAMs).

In order to achieve the successful fabrication of FT-RRAMs, a flexible transparent electrode needs to be developed that fully satisfies the following requirements: (1) it should be transparent and have electrical conductive properties comparable to indium tin oxide (ITO) deposited on glass; (2) it needs to be mechanically stable, so that it can resist severe mechanical deformations; (3) the surface should be sufficiently smooth to prevent the formation of leakages through the thin TiO_2_ layer; and (4) the electrode should have physical contact with the overlapping TiO_2_ layer, through abundant pathways, to bring up the advantages of TiO_2_-based RRAMs^12–24^. A percolated network of silver nanowires (AgNWs) have been reported as a powerful choice for flexible electrodes, mainly because of its high figure-of-merit (the electrical to optical conductivity ratio) and high intrinsic flexibility^25–28^. For instance, recent works by Park *et al*.^25^ and Yang *et al*.^26^ employed the AgNWs, and treated with flash light to induce the self-limited plasmonic welding. The plasmonically welded network of AgNWs was highly conductive, transparent, mechanically stable and can have abundant physical contact with the overlying layer. According to Xiong *et al*., highly conductive and air-stable AgNW electrodes could be achieved by electroless-welding of AgNW network with an overcoating of an iongel layer^27^. Wu *et al*. fabricated polyester/AgNW/graphene core-shell nanocomposites to achieve the transparent electronic textile that can be used in wearable electricity generator, but most importantly^28^, all those studies did not resolve the issue with the rough surface of the AgNW-based electrodes^25–28^.

AgNW networks embedded in the surface of colorless polyimide (cPI) was considered a good candidate for flexible and transparent electrode of FT-RRAMs due mainly to high figure-of-merit, high mechanical stability even under an extreme bending sequence, and smooth surface comparable to that of ITO on glass^29, 30^. However, a single issue remains, in that the surface coverage of the AgNWs is quite small, and thin layers of the cPI cover the AgNWs embedded in the surface of the composite electrode. In our previous study, although a plasma treatment of the AgNWs/cPI surface was revealed to be effective in enlarging the conductive pathways to the overlying layers, the electrode’s surface was being roughened by anisotropic polymer etching, which can induce leakage current^29^. A new approach is essentially needed so that the AgNWs/cPI composite electrode can be used as a FT-RRAMs transparent electrode, without deteriorating its smooth surface.

Herein, we propose a new approach to fabricate a AgNWs/cPI composite electrode that concurrently has a high figure-of-merit, mechanical stability, smooth surface, and abundant coverage of AgNWs at its surface. For this, a simple plasma treatment was applied to the AgNWs, which had been previously deposited on a glass substrate, before embedding them in the surface of the cPI, which was found to enlarge the contact areas between the nanowires and the glass. An inverted layer-processing method with an additional solvent treatment was used to embed the treated AgNWs in the cPI surface. The fabricated composite electrode was used to make a FT-RRAM, which comprised of a simple configuration of Pt/TiO_2_/AgNWs/cPI. In conventional method, organic materials were generally used for achieving the flexible resistive switching due to flexibility, easy-to-fabrication, lightweight, and large-area processibility, therefore, a number of researches about organic-based resistive switching memory have been performed by various groups^31–34^. Compared with these organic-based resistive switching memory, our devices showed very competitive characteristics for flexible and transparent electronics, in that the average low operation voltage^31–34^, on-off ratio^33^, and retention time^34^ were measured to be 1 V for the SET process and −0.6 V for the RESET process, about 200 at −0.2 V, and longer than 10^6^ s, respectively.

## Methods

### Fabrication of the FT-RRAM device

**Figure 1:**
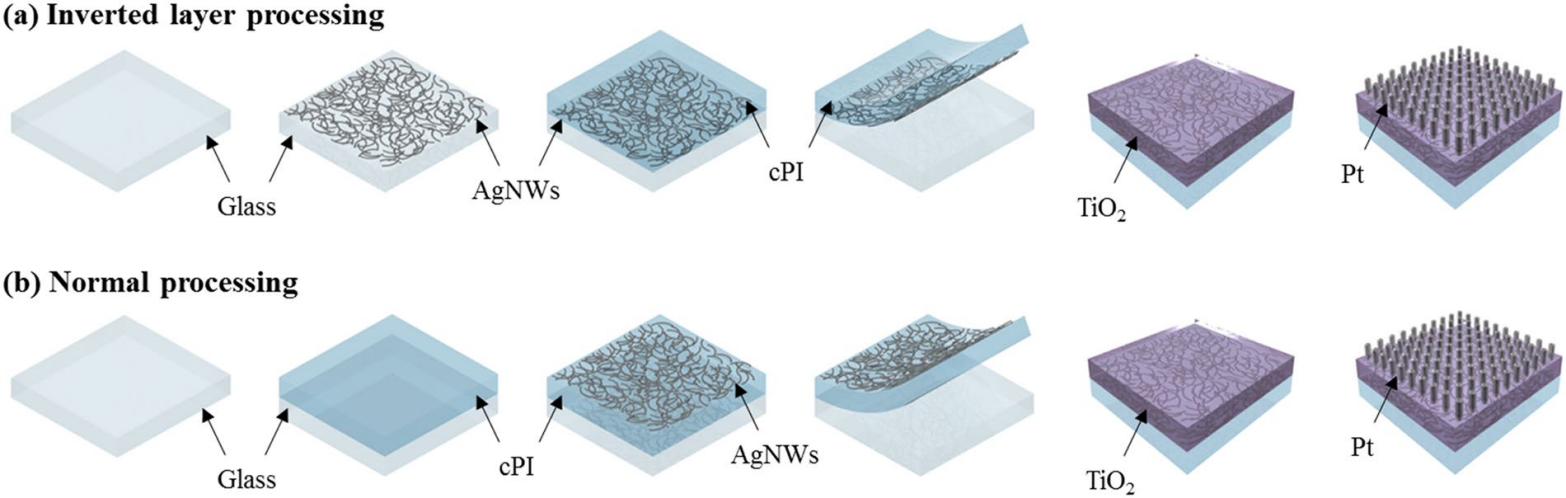
Schematic of the fabrication procedure for AgNWs/cPI-based FT-RRAMs: (**a**) inverted layer processing and (**b**) normal processing.

The fabrication procedure of the FT-RRAMs is schematically illustrated in Fig. 1. Two separate procedures were employed to prepare the AgNWs-based electrodes: the first one being the inverted layer processing procedure shown in Fig. 1a, and the other a normal electrode processing approach summarized in Fig. 1b. For the inverted layer processing, a solution of AgNWs dispersed in isopropanol (Nanopyxis Ltd., Korea) was first spin-coated on a glass substrate, followed by infrared (IR) irradiation for 10 min to remove the remaining solvent. The average diameter and length of the nanowires were around 35 nm and 30 μm, respectively. A treatment with Ar plasma was employed for 10 min at a power density of 8 W/cm^2^ to collapse the nanowires onto the glass by causing their partial melting. For the plasma treatment, the gas flow rate and gas pressure were controlled to be 40 ml/min and 20 Pa, respectively. A cPI varnish (Kolon Industries, INC., Korea) was spin-coated onto the glass/AgNWs, and the system was subsequently cured at 200 °C for 1 h to form a cPI film with a thickness of about 20 μm. To enhance the wettability of the cPI varnish, the electrode was first treated with N-Methyl-2-pyrrolidone (NMP) before being coated with the varnish. Once the cPI film was formed on the composite electrode, the sample was soaked in water for 10 min to induce hygroscopic swelling of the cPI film, so as to allow the film to be safely peeled off from the supporting glass. For the normal coating approach, the processing sequence of the AgNW dispersion and cPI varnish coat were switched, so that the AgNWs would be deposited onto the cPI film, as shown in Fig. 1b. To investigate the exposure of nanowires on the composite electrodes, the fabricated AgNWs-based electrode underwent electroless plating for 10 min at 85 °C in a bath containing 7 g/L of CuSO_4_‚ 5H_2_O, 25 g/L of potassium sodium tartrate, 4.5 g/L of sodium hydroxide, and 9.5 g/L of formaldehyde. In order to complete the FT-RRAM, a TiO_2_ thin film was deposited onto the AgNWs/cPI electrode, to formulate the resistive switching material, by employing an atomic layer deposition (ALD) system. A detailed method of the ALD process is explained in the Supplementary Information. Finally, the 100-nm Pt top electrode with a diameter of 250 μm was sputtered by a radio frequency (RF) sputtering system.

### Characterization of the AgNWs/cPI electrode and FT-RRAM device

A scanning electron microscope (SEM; S-4800, Hitachi, Japan) was used to investigate the microstructure of the AgNW networks. The optical transmission was also measured using a UV–visible spectrophotometer (V-560, Jasco, Japan), while the sheet resistance (R_s_) was measured using a non-contact measurement system (EC-80P, Napson Corporation, Japan). The surface morphology was measured using atomic force microscopy (AFM; XE-100TM, Park Systems, USA). The mechanical stability of the film was evaluated using an automatic bend-testing machine (Bending tester, Jaeil Optical Systems, Korea), whereby a bending radius of 0.5 mm was used to induce a ~2% outward strain. The films were bent at a cycle rate of 0.3 Hz, with their resistance being measured during the bending cycles. A tape test was performed to investigate adhesion between the AgNWs and the cPI by employing a commercial Kapton tape. For characterizing the FT-RRAM devices, the electrical properties of the FT-RRAM were measured by a semiconductor characterization system (4200-SCS, Keithley, USA). During the measurements, a bias was applied to the bottom electrode (AgNWs) while the top electrode was grounded. All measurements were performed at 25 °C in air with no light conditions. After the measurement, the devices were stored in vacuum container to avoid moisture and oxygen for the best device conditions. One more bending test was conducted to evaluate the flexibility of the devices using a bending chuck designed to induce a curvature radius of 10 mm.

## Results and Discussion

**Figure 2:**
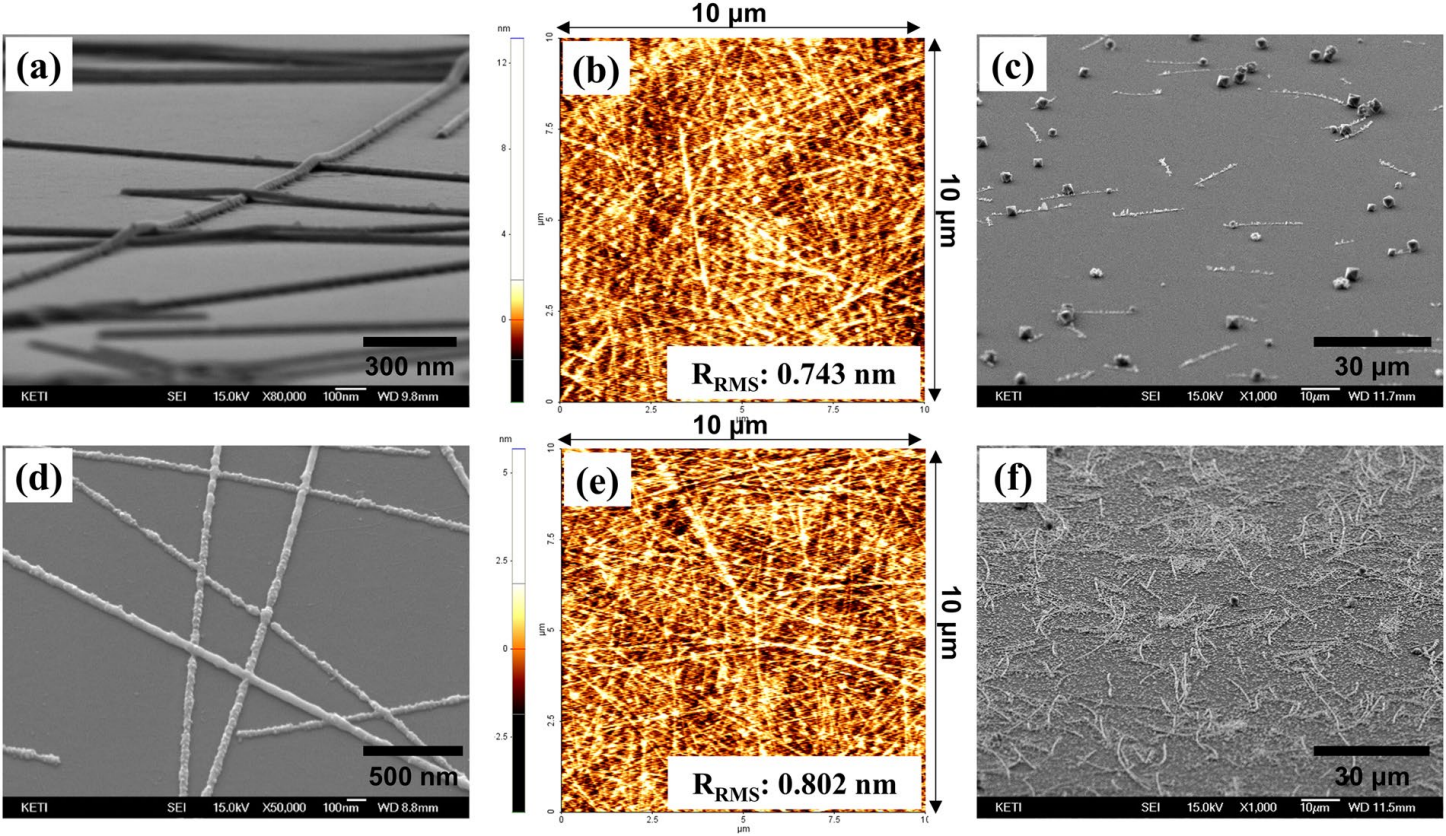
(**a**) SEM micrograph of AgNWs on a glass substrate without an Ar plasma treatment, (**b**) AFM surface micrograph of an untreated AgNWs/cPI electrode, (**c**) SEM micrograph of the untreated AgNWs/cPI electrode after immersion into a Cu plating solution, (**d**) SEM micrograph of AgNWs on a glass substrate after an Ar plasma treatment, (**e**) AFM surface micrograph of a treated AgNWs/cPI electrode, and (**f**) SEM micrograph of the treated AgNWs/cPI electrode after immersion into a Cu plating solution.

First, a AgNW dispersion was coated on a glass substrate to form a percolated conductive network as shown in Fig. 2a. In that case, the AgNWs were only placed on the surface of the glass substrate, and not firmly adhered to it. Some gaps definitely formed, and were observable, at the interface between the AgNWs and the glass. Therefore, the nanowires could be easily peeled off or damaged, even by weak external stresses such as bending, rubbing, or scratching. In our previous studies, we developed an inverted layer-processing technique to bury the nanowires into the surface of a transparent polymer (cPI), resulting in an AgNWs/polymer composite electrode^29, 30^. By this approach, an extremely smooth surface could be obtained, as shown in Fig. 2b, because most of the nanowire bundles were buried underneath the surface of the cPI. This perfect embedment originated from the fact that the cPI varnish was completely diffused and could infiltrate the nanopores formed between the nanowires and glass substrate. High mechanical stability was another powerful merit that could be obtained by this method. However, a serious problem with the fabricated electrodes remained: a limited coverage of the conductive pathways to the overlying layer. In order to investigate the areas of exposed nanowires on the AgNWs/cPI composite, we immersed the composite electrodes in a Cu electroless plating solution without employing any preprocessing such as forming seed materials on the surface of the samples. Electroless plating is an autocatalytic chemical method mainly used to deposit a layer of specific metals, such as Cu, Ni and Au, on a metal or polymer. This approach relies on the presence of a reducing agent, which reacts with the metal ions to reduce them and deposit the metal. Inevitably, Cu was metallized onto very limited areas on the surface of AgNWs/cPI as shown in Fig. 2c, revealing the constrained nanowire-exposure. In our previous study, a plasma treatment onto the electrode proved to be effective for enlarging the nanowire exposure, but in that case, the surface morphology could be roughened^29^.

Here, we applied the Ar plasma treatment for 10 min, in a different sequence, onto the nanowires that had been deposited on the glass substrate (shown in Fig. 2a) to collapse them. The treatment significantly changed the morphology of the AgNW networks as shown in Fig. 2d, as if they had been melted by the treatment. It is interesting to note that the nanowires were not seriously damaged by the excessive melting in terms of their interconnection. The pores that existed at the interface between the AgNWs and glass substrate were not observed after the plasma treatment, implying that the contact areas between them were enlarged by the treatment, possibly owing to their partial melting and wetting to the substrate. This is noteworthy because an enlarged contact area should provide abundant exposure of the nanowires for better contact with the overlying layer. The nanowires would then be embedded in the surface of the polymer by first coating them with the polymer, followed by curing and peeling-off (Fig. 1a). In a precedent work from our group, an analysis with X-ray photoelectron spectroscopy (XPS) revealed that a thin insulation layer of polyvinylpyrrolidone (PVP) could be successfully removed by the Ar plasma treatment^35^. In this study, we could also investigate that the R_s_ of the AgNW electrode decreased by about 30% after the Ar plasma treatment. Unfortunately, overcoating the treated AgNWs with cPI, followed by curing, did not lead to their perfect embedment. Around 5 nm of roughness in root mean square (R_RMS_) was measured on the surface of the electrode, which is larger than that of sputter-derived commercial ITOs deposited on a glass substrate, or the one shown in Fig. 2b. In the no-treatment case, the carbonyl groups of PVP surrounding the AgNWs presumably form hydrogen bonds with the carboxylic and amide groups of the cPI, which could enhance their adhesion and wettability. The absence of PVP caused the incomplete infiltration of the cPI varnish into the plasma treated nanowire intersections and narrowed gaps between the AgNWs and glass substrate.

To resolve this issue, the plasma treated AgNWs in Fig. 2d were treated with DMA, which is a solvent of the cPI varnish. An interesting finding from this treatment was that the wettability of the cPI varnish to the electrode was significantly enhanced, presumably due to an effect of the pretreatment. The wetting of the highly viscous varnish could be improved by pretreating it with a low viscosity solvent, resulting in the perfect infiltration of the cPI varnish into the pores between the AgNWs and glass substrate. A study to reveal the mechanism for this improvement is currently ongoing. The smoothened surface of the embedded electrodes is shown in Fig. 2e, which is comparable to that of the non-treated electrodes in Fig. 2b. The main effect of the plasma treatment is shown in Fig. 2f, in that the Cu was very densely plated on the exposed AgNWs after the electrode was immersed in the Cu plating solution, proving the enlargement of the nanowire exposure. This implies that extremely smooth surfaces and abundant nanowire coverage could be simultaneously achieved by this simple approach.

**Figure 3:**
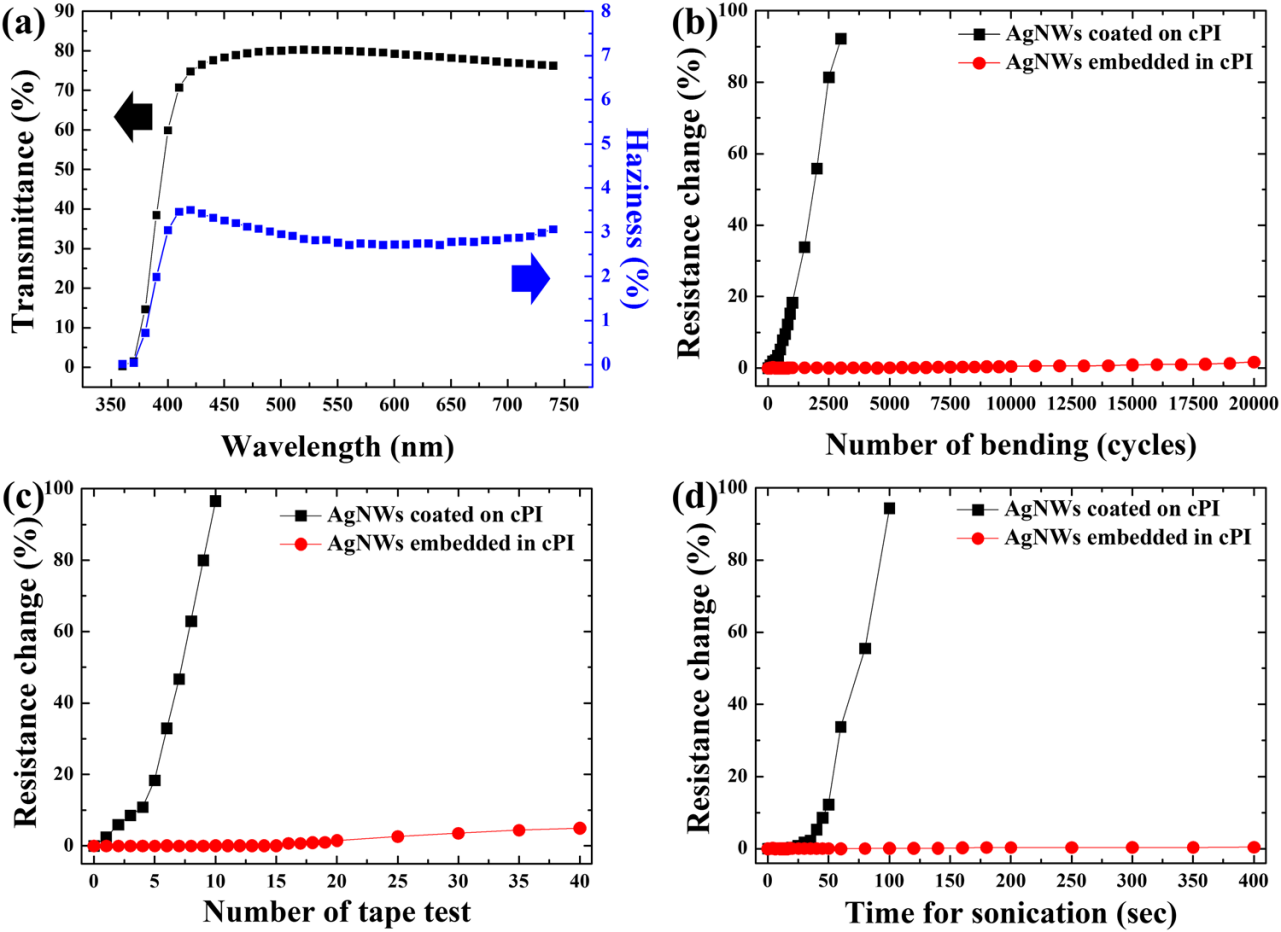
(**a**) Transmittance and haziness of the AgNWs/cPI electrode, (**b**–**d**) resistance change of the AgNWs/cPI electrodes as a function of number of bending cycles, tape tests, and sonication time, respectively.

Figure 3a shows the transmittance and haziness (i.e. the ratio of diffused to total transmission) of the composite electrode, which was optimized to have an R_s_ of approximately 5.5 ohm/sq. Over a very broad spectral range of 420–700 nm, the electrode exhibited a high transmittance of more than 80%, which implies that its performance is better than that of the commercially available ITO deposited on polyethylene terephthalate (PET). Additionally, the haziness of the electrode was less than 3% over the spectral range, implying that the electrode is optically clear. The mechanical stability of the electrodes was evaluated by employing various tests such as bending, taping, and ultra-sonication. For comparison, the AgNWs that were directly deposited on the cPI, produced using the procedure in Fig. 1b, were tested as well. First, a cyclic bending test was evaluated by producing a tensile strain of about 2%. Figure 3b shows the change in resistance (%) as a function of the number of bending cycles. The resistance of the embedded electrodes was virtually unaffected during the 20,000 bending cycles, which means that the mechanical stability of the fabricated electrodes did not severely deteriorate. Surface-coated electrodes, on the other hand, exhibited a fast increase in resistance, of approximately 100% after 3000 bending cycles, mainly due to poor adhesion of the AgNWs to the cPI. The adhesion test, with repeated taping and releasing, revealed that the AgNWs embedded in the cPI were very robustly adhered to the cPI as shown in Fig. 3c. Moreover, the resistance of the embedded electrodes was not deteriorated even after being immersed in an ultra-sonication bath for up to 400 s (Fig. 3d). To the best of our knowledge, this is the first example achieving most of the requirements for the fabrication of flexible transparent electrodes, i.e. a high figure-of-merit, smooth surface, mechanical stability, and abundant surface coverage of the conductive medium.

**Figure 4:**
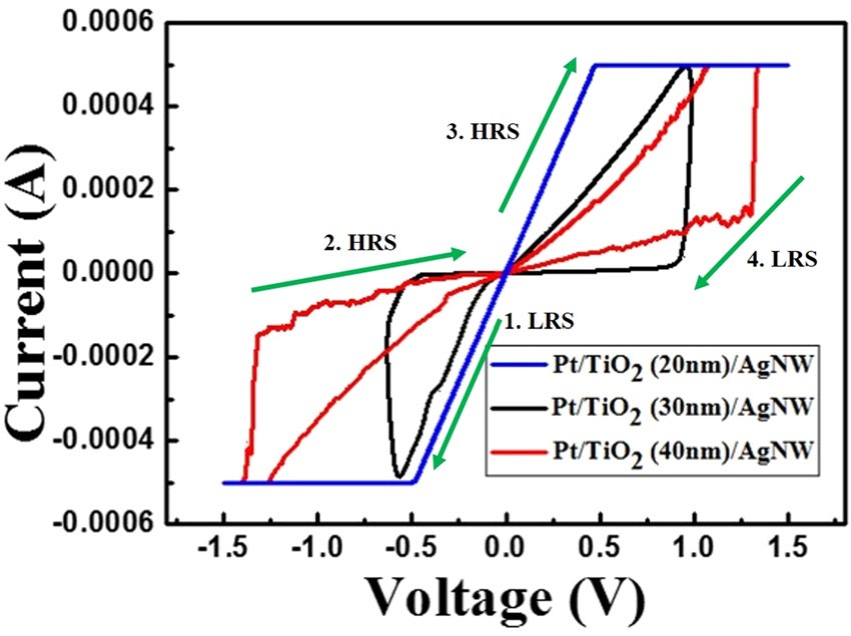
Typical bipolar I–V curve of the FT-RRAM device with different TiO_2_ insulator thickness.

Based on our experiments with the AgNWs/cPI composite electrode, we fabricated the FT-RRAM. Figure 4 shows the current-voltage (I–V) characteristics of the Pt/TiO_2_/AgNW memory cell, which was obtained by DC voltage sweep measurements using different thickness of the active layer so as to optimize the oxide thickness. For all measurements, the Pt electrodes were grounded, a bias was applied to the AgNW electrodes, and a compliance current of 500 μA was imposed during the forming and set processes to prevent breakdown of the device by the abrupt increase in current during switching. Depending on the TiO_2_ thickness, different switching characteristics were observed. In the case of a 20 nm TiO_2_ thickness, upon sweeping, the FT-RRAM shows Ohmic behaviour in all sweeping regions. This indicates that the thin oxide film of TiO_2_ causes short-circuit of the FT-RRAM. In the case of a 30 nm TiO_2_ thickness, a forming process was essential before operating the FT-RRAM device, because the device was initially in the high resistance state (HRS) of the as-deposited condition. When a positive bias voltage (0 to 2.5 V) was applied to the AgNW electrode, a rapid increase in the current was observed at roughly 1.6 V (V_Forming_), as shown in Figure S1. The forming process was essential for initiating the FT-RRAM, so that device would convert from the HRS to a low resistance state (LRS). Subsequently, the I–V properties of the FT-RRAM structure were determined by voltage sweep measurements in the sequence 0 V → −0.65 V → 0 V → 1 V → 0 V. After the forming process, when a negative voltage was applied to the AgNW electrode of the FT-RRAM, in the LRS, the device went back to the HRS at a specific negative voltage (VRESET). This transition is the reset process. When a positive voltage was applied to the AgNW electrode in the HRS, resistive switching back to the LRS occurred at a specific positive voltage (VSET). This transition is the set process. In this work, the FT-RRAM usually had a V_SET_ of 1 V for switching from the HRS to the LRS, and a V_RESET_ of −0.6 V for switching from the LRS to the HRS. Lastly, in the case of a 40 nm TiO_2_ thickness, the FT-RRAMs also showed bipolar resistive switching characteristics. However, higher set and reset voltages than those for a 30 nm TiO_2_ thickness were measured that cause high power consumption making the FT-RRAM unreliable. In addition, in case of FT-RRAM with 30 nm thickness of TiO2, they show good I–V reliability as can be inferred by endurance and retention tests in the later part of this manuscript. Therefore, the optimized FT-RRAM with a 30 nm TiO_2_ thickness showed the best performance to be used as an active material for our resistive switching memory.

Additionally, we also measured the transmittance characteristics. As shown in Figure S2, the optical transmittance spectra of the FT-RRAM obtained using a UV-visible spectrophotometer was measured. The average transmission in the visible region (400–800 nm in wavelength) of the FT-RRAM, including the substrate, is approximately 75.6%. This semi-transparent characteristic is due to the transparent AgNW electrodes of the device, the wide band gap of the thin TiO_2_ layer, pillar-patterned Pt electrodes and the transparent cPI substrate. The transmission of the device is slightly lower because we used Pt electrode as the top electrode to maximize the memory characteristics for cation migration-based RRAMs^36, 37^, but it can be improved by using transparent conductive electrode as the top electrode for anion-based memory. Nevertheless, our device compared with the previous fabricated transparent RRAM device, showed higher transmission^6^, better endurance and longer retention^7^, low-voltage operation^8^, even characteristic of flexibility with simple fabrication^9^. Therefore, we believe that this result can be immediately applied to a transparent RRAMs.

**Figure 5:**
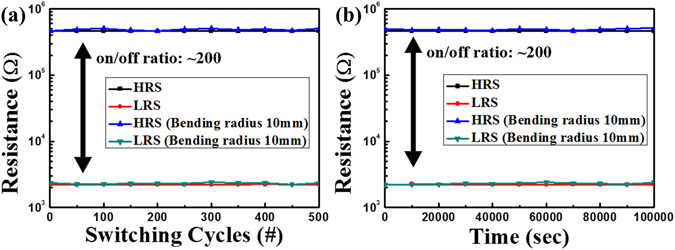
(**a**) Endurance and (**b**) retention characteristics of the FT-RRAM at flat and bent states, respectively, measured at a V_READ_ = −0.2 V.

For further analysis, the endurance and retention were measured, including the bent state, using a bending chuck with a curvature radius of 10 mm to examine the mechanical stability, reliability, and non-volatility of the FT-RRAM. Figure S3 shows the measurement set-up. The reading voltage after the set and reset processes was −0.2 V in both tests. As can be seen in Fig. 5a, no significant changes in the resistance magnitudes for 500 cycles are observed, even at the bent state showing on/off ratio of over 200. This endurance test confirmed the excellent reliability of our device even under bent states. Furthermore, the retention test was also examined in both the flat and bent state (extended range of the retention and endurance are shown in Figure S4). As shown in Fig. 5b, the current was measured every 10^4^ s. The resistances in both states, under the bent state, fluctuated slightly. However, the fluctuations of the resistances were sufficient to dissolve both states. These retention results show that the FT-RRAM has excellent non-volatility. The results of the endurance and retention tests indicate that the performance of our FT-RRAM is not affected by acute bending of the device. In addition, we tested 16 samples for the reliability of our devices summarized in Figure S5 and Table 1. At the point of view about resistance ratio, in the case of MRAM, the resistance ratio of only 1.2 to 1.3 can be utilized by exquisite circuit design^1, 2^. Therefore, resistance ratio of our FT-RRAM devices is enough to effectively distinguish by using today’s highly sense amplifiers. Furthermore, it can be improved by various methods such as embedding Ag nano particles into the insulating medium and applying higher compliance current^38, 39^. Even for mass production of FT-RRAMs, small on/off ratio issues will be solved by computational program such as error correction code (ECC) and systematic fabrication process.

**Figure 6:**
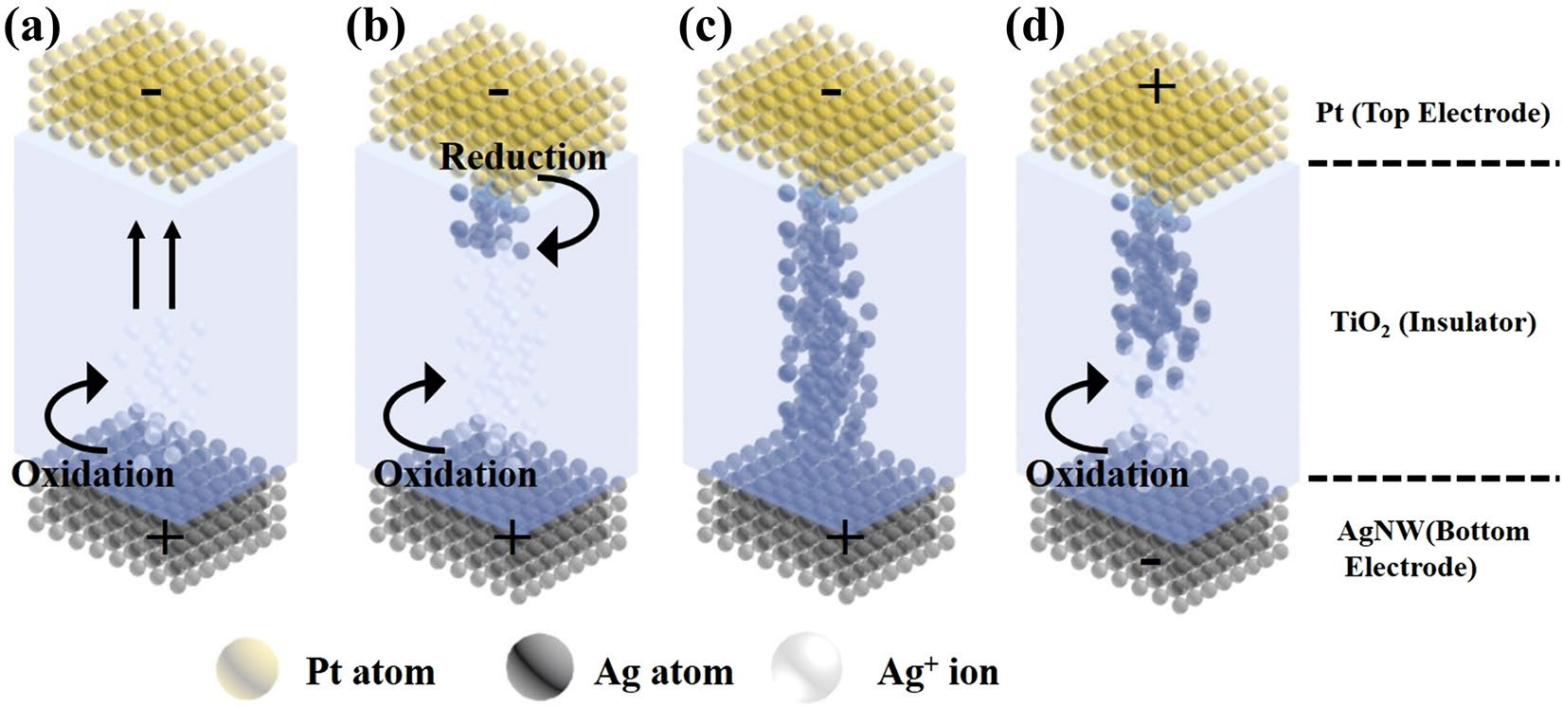
Schematics of the resistive switching process in the FT-RRAM cell.

Figure 6 illustrates a schematic diagram of the cation-based resistive switching mechanism for our FT-RRAM device (organized mechanism and switching processes are summarized in provided in Supplementary Information). When a positive voltage is applied to the AgNW electrode (active electrode), an oxidation process of the Ag atoms occurs and Ag^+^ cations are generated, which could be described as Ag → Ag^+^  + e^−^ (Fig. 6a). The mobile Ag^+^ cations move toward the Pt electrode (inert electrode) through the TiO_2_ layer, and are reduced by electrons injected from the Pt electrode i.e. Ag^+^  + e^−^ → Ag (Fig. 6b). The consecutive sedimentation of Ag metal atoms on the Pt electrode leads to the growth of Ag filaments, which when they reach the AgNW electrode form highly conductive filaments in the ON state (Fig. 6c). When a negative voltage is applied to the AgNW electrode, an electrochemical dissolution takes place somewhere along the conductive filaments and causes a reset process (Fig. 6d)^2, 40–42^.

## Conclusion

Highly flexible and transparent electrodes comprising of a percolated network of AgNWs embedded at the surface of cPI were fabricated. By employing a plasma treatment to collapse the nanowires on a preliminary glass substrate, the coverage of the conductive pathways at the surface of the AgNWs/cPI electrode could be significantly enlarged. Furthermore, a pretreatment with DMA improved the wettability of the cPI varnish onto the plasma treated AgNWs, resulting in an extreme smoothness of the composite electrode. As a result, for the first time, four important requirements for the successful employment of the electrodes in the fabrication of thin film based devices were simultaneously satisfied: a high figure-of-merit (around 700–800), mechanical stability (curvature radius of 0.5 mm), surface smoothness (R_RMS_ less than 1 nm), and abundant surface coverage of the conductive networks (much of the nanowires were exposed to air). The fabricated AgNWs/cPI electrodes were used as a bottom electrode and substrate to construct the FT-RRAMs. The device successfully shows flexible and semi-transparent resistive switching memory characteristics including high on/off ratio, excellent endurance, and long retention times even at the bent state. We tried to explain the cation-based resistive switching mechanism to interpret our memory system. To the best of our knowledge, this is the first example of AgNW-based electrodes used in the fabrication of RRAMs. We are convinced that our FT-RRAM device will bring a technology breakthrough and achieve success in future flexible and transparent electronic devices.

## Electronic supplementary material

Supplementary Information

## References

[1] Waser R, Aono M (2007). Nanoionics-Based Resistive Switching Memories. Nat. Mater..

[2] Waser R, Dittmann R, Staikov C, Szot K (2009). Redox-Based Resistive Switching Memories Nanoionic Mechanisms, Prospects, and Challenges. Adv. Mater..

[3] Valov I, Kozicki MN (2013). Cation-Based Resistance Change Memory. J. Phys. D: Appl. Phys..

[4] Yang Y, Lu W (2013). Nanoscale Resistive Switching Devices: Mechanisms and Modeling. Nanoscale.

[5] Yang YC (2009). Fully Room-Temperature-Fabricated Nonvolatile Resistive Memory for Ultrafast and High-Density Memory Application. Nano Lett..

[6] Zou L, Hu W, Xie W, Bao D (2017). Uniform Resistive Switching Properties of Fully Transparent TiO_2_-Based Memory Devices. J. Alloy. Comp.

[7] Rani A (2016). Non-Volatile ReRAM Devices Based on Self-Assembled Multilayers of Modified Graphene Oxide 2D Nanosheets. Small.

[8] Qian K, Nquyen VC, Chen T, Lee PS (2016). Novel Concepts in Functional Resistive Switching Memories. J. Mater. Chem. C.

[9] Qian K, Cai G, Nguyen VC, Chen T, Lee PS (2016). Direct Observation of Conducting Filaments in Tungsten Oxide Based Transparent Resistive Switching Memory. ACS Appl. Mater. Interfaces.

[10] Hsiung CP (2010). Formation and Instability of Silver Nanofilament in Ag-Based Programmable Metallization Cells. ACS Nano.

[11] Kwon D-H (2010). Atomic Structure of Conducting Nanofilaments in TiO2 Resistive Switching Memory. Nat. Nanotechnol..

[12] Wang H, Zhou D, Cao J (2013). Development of a Stretchable Conductor Array With Embedded Metal Nanowires. IEEE Trans. Nanotechnol..

[13] Zhu R (2011). Fused Silver Nanowires with Metal Oxide Nanoparticles and Organic Polymers for Highly Transparent Conductors. ACS Nano.

[14] Guo H (2013). Copper Nanowires as Fully Transparent Conductive Electrodes. Sci. Rep.

[15] Won Y (2014). Annealing-Free Fabrication of Highly Oxidation-Resistive Copper Nanowire Composite Conductors for Photovoltaics. NPG Asia Mater.

[16] Nirmalraj PN (2012). Manipulating Connectivity and Electrical Conductivity in Metallic Nanowire Networks. Nano Lett..

[17] Van GJ, Spinelli P, Polman A (2012). Transparent Conducting Silver Nanowire Networks. Nano Lett..

[18] Liang J, Li L, Niu X, Yu Z, Pei Q (2013). Elastomeric Polymer Light-Emitting Devices and Displays. Nat. Photonics.

[19] Ho X (2013). Biaxially Stretchable Silver Nanowire Transparent Conductors. J. Appl. Phys..

[20] Yim JH (2014). Fully Solution-Processed Semitransparent Organic Solar Cells with a Silver Nanowire Cathode and a Conducting Polymer Anode. ACS Nano.

[21] Ahn Y, Lee H, Lee D, Lee Y (2014). Highly Conductive and Flexible Silver Nanowire-Based Microelectrodes on Biocompatible Hydrogel. ACS Appl. Mater. Interfaces.

[22] Tao Y (2013). High-Reproducibility, Flexible Conductive Patterns Fabricated with Silver Nanowire by Drop or Fit-to-Flow Method. Nanoscale Res. Lett..

[23] Kumar ABVK, Bae CW, Piao L, Kim SH (2013). Silver Nanowire Based Flexible Electrodes with Improved Properties: High Conductivity, Transparency, Adhesion and Low Haze. Mater. Res. Bull..

[24] Kumar ABVK (2014). Silver Nanowire/polyaniline Composite Transparent Electrode with Improved Surface Properties. Mater. Res. Bull..

[25] Park, J. H. *et al*. Flash-Induced Self-Limited Plasmonic Welding of Silver Nanowire Network for Transparent Flexible Energy Harvester. *Adv. Mater*. doi:10.1002/adma.201603473. 10.1002/adma.201603473 27892631

[26] Yang Y (2016). Facile fabrication of stretchable Ag nanowire/polyurethane electrodes using high intensity pulsed light. Nano Res..

[27] Xiong W (2016). Highly Conductive, Air-Stable Silver Nanowire@Iongel Composite Films toward Flexible Transparent Electrodes. Adv. Mater..

[28] Wu X, Kim TW, Li F, Guo T (2016). Wearable Electricity Generators Fabricated Utilizing Transparent Electronic Textiles Based on Polyester/Ag Nanowires/Graphene Core-Shell Nanocomposites. ACS Nano.

[29] Ok K-H (2015). Ultra-Thin and Smooth Transparent Electrode for Flexible and Leakage-Free Organic Light-Emitting Diodes. Sci. Rep.

[30] Kim Y (2015). Inverted Layer-By-Layer Fabrication of an Ultraflexible and Transparent Ag Nanowire/Conductive Polymer Composite Electrode for Use in High-Performance Organic Solar Cells. Adv. Funct. Mater..

[31] Nau S, Wolf C, Sax S, List-Kratochvil JW (2015). Organic Non-Volatile Resistive Photo-Switches for Flexible Image Detector Arrays. Adv. Mater..

[32] Busby Y (2015). Direct Observation of Conductive Filament Formation in Alq3 Based Organic Resistive Memories. Appl. Phys. Lett..

[33] Casula G, Cosseddu P, Bonfiglio A (2015). Integration of an Organic Resistive Memory with a Pressure-Sensitive Element on a Fully Flexible Substrate. Adv. Ener. Mater.

[34] Ma L (2003). Nonvolatile Electrical Bistability of Organic/Metal-Nanocluster/Organic System. Appl. Phys. Lett..

[35] Kim DG (2016). Electrically and Mechanically Enhanced Ag Nanowires-Colorless Polyimide Composite Electrode for Flexible Capacitive Sensor. Appl. Surf. Sci..

[36] You BK (2016). Reliable Memristive Switching Memory Devices Enabled by Densely Packed Silver Nanocone Arrays as Electric-Field Concentrators. ACS Nano.

[37] Xiao B, Yu X-F, Cheng J-B (2016). Atomic Insight into the Origin of Various Operation Voltages of Cation-Based Resistance Switches. ACS Adv. Mater. Intefaces.

[38] Au K (2013). Enhanced Resistive Switching Effect in Ag Nanoparticle embedded BaTiO3 Thin Films. J. Appl. Phys..

[39] Mutiso RM, Kikkawa JM, Winey KI (2013). Resistive Switching in Silver/Polystyrene/Silver Nano-Gap Devices. Appl. Phys. Lett..

[40] Pan F (2014). Recent Progress in Resistive Random Access Memories: Materials, Switching Mechanisms, and Performance. Mater. Sci. Eng. R Reports.

[41] Valov I, Waser R, Jameson JR, Kozicki MN (2011). Electrochemical Metallization Memories–Fundamentals, Applications, Prospects. Nanotechnology.

[42] Zhuge F (2015). Mechanism for Resistive Switching in Chalcogenide-Based Electrochemical Metallization Memory Cells. AIP Adv..

