# Supplementary material for: Silver Nanowire/Colorless-Polyimide Composite Electrode: Application in Flexible and Transparent Resistive Switching Memory
Source: Sci Rep. 2017 Jun 13;7:3438. doi: 10.1038/s41598-017-03746-1 (PMC5469806; doi:10.1038/s41598-017-03746-1)
Supplement: Supplementary file 1 — Supplementary Information [file 41598_2017_3746_MOESM1_ESM.pdf]

Supplementary Information

Silver Nanowire/Colorless-Polyimide Composite Electrode: Application in Flexible and Transparent Resistive Switching Memory

Seung-Won Yeom, Banseok You, Karam Cho, Hyun Young Jung, Junsu Park, Changhwan Shin, Byeong-Kwon Ju and Jong-Woong Kim

ALD Process

In the ALD process, ALD TiO_2 was synthesized using titanium tetraisopropoxide (TTIP) as a precursor and deionized water as an oxidant, respectively. The titanium precursor was heated to 50 ^{o}C, and the water was evaporated at 25 ^{o}C. The deposition temperature was 150 ^{o}C, and the precursor delivery lines were heated to 90 ^{o}C. The pulse times of TTIP and distilled water were set at 0.1 s and 0.2 s, respectively. Dry nitrogen was used as the purge gas with a flow rate of 200 sccm. In one ALD cycle, H_2O + N_2 gas were fed into the reactor chamber. Then, N_2 purging was carried out. The purging time between the precursor and the oxidant was 18 s. For the deposition of a 20/30/40-nm-thick TiO_2 film, 800/1200/1600 cycles of ALD were performed, respectively. The base pressure of the chamber with the nitrogen gas flow was maintained at approximately 0.9 Torr, and the chamber pressure during the precursor pulsing was approximately 0.9 Torr.

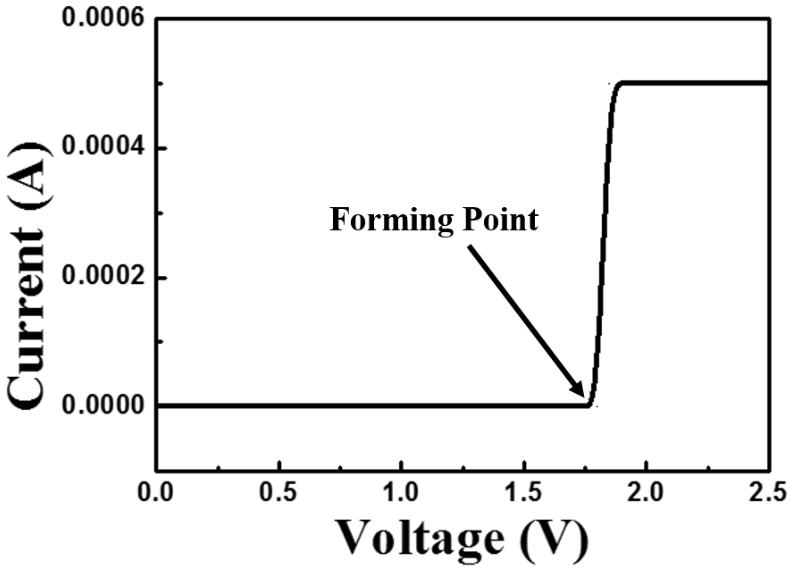

Figure S1. Forming process of the FT-RRAM consisting of a Pt/TiO_2/AgNW cell.

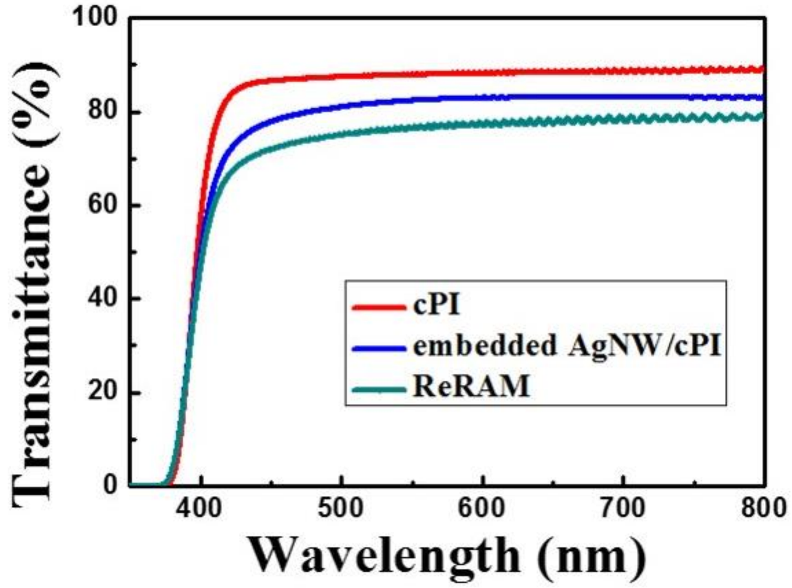

Figure S2. Optical transmission spectrum of the cPI substrate, embedded AgNW/cPI substrate, and FT-RRAM.

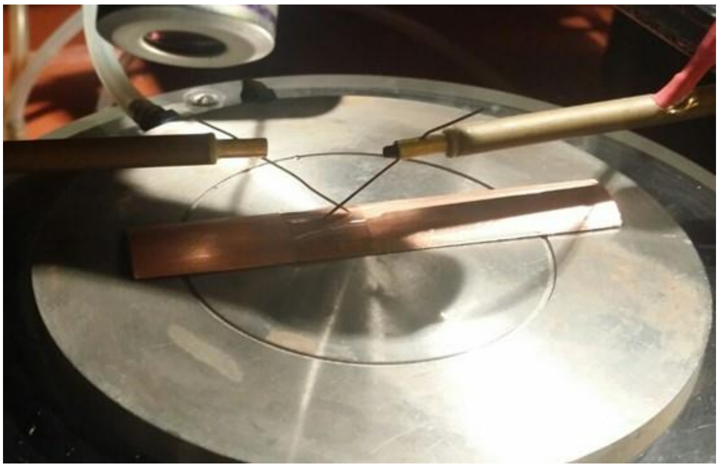

Figure S3. The measurement set-up for a bending test of the fabricated FT-RRAM.

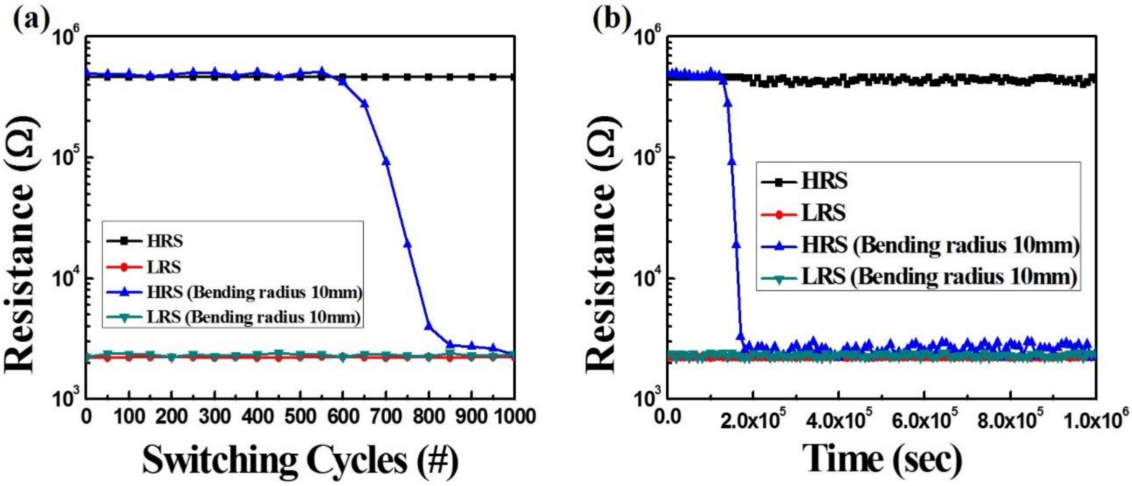

Figure S4. Extended (a) endurance and (b) retention tests. The HRS under bent states in both graphs are decreased after 600 cycles, 10^5 sec, respectively. This is caused by that switching filament in the FT-RRAM is not effectively ruptured by bent state. But these data are competitive compared with previous flexible resistive switching memories [1-4].

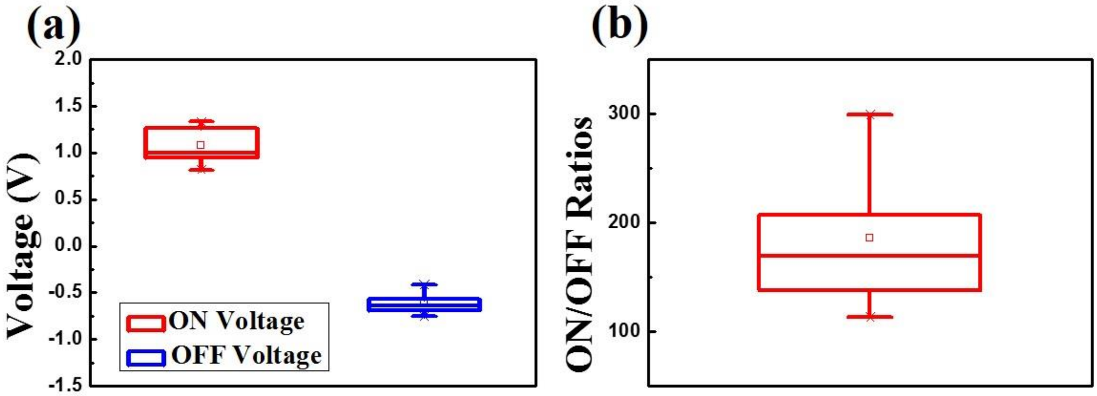

Figure S5. (a) operating voltages and (b) ON/OFF ratios averaged over 16 samples.

	Operating Voltage for the SET	Operating Voltage for the RESET	ON/OFF Ratios
Averaged	1.076 V	-0.609 V	185.78
Maximum	1.333 V	-0.75 V	299.53
Minimum	0.819 V	-0.411 V	113.09

Table 1. Summary of the averaged, maximum, minimum operating voltage for the SET and RESET process and ON/OFF ratios.

Resistive switching processes in FT-RRAM

Cation-based RRAM basically uses an oxidizable positive electrode (anode) such as Ag or Cu that generally acts as ion source to maintain ion concentration and overall charge neutrality. An inert counter negative electrode such as W, TiN, Pt act as electron source to reduce the cation for growth of filament in oxide medium. And, solid electrolyte and oxide-materials between the electrodes serves to transport the metal cations.

In our device, nanoionic and electrochemical redox are essential point to understand the mechanism. The net processes of the resisitve switching are organized below;

A SET process occurs if an applied voltage is greater than the oxidation potential of the Ag NW.

1. Anodic dissolution of Ag according to oxidation of AgNW (Figure 6(a))

   $Ag \rightarrow Ag^{+} + e^{-}$

   Ag^{+} is most common oxidation state in the TiO_2 film.
2. Drift of the Ag+ cations via the TiO_2 film by an applied high electric field; (Figure 6(a))
3. Reduction and sedimentation of Ag metal atoms on the surface of the Pt growing preferentially toward AgNW electrode. (Figure 6(b))

   $Ag^{+} + e^{-} \rightarrow Ag$;
4. Growth of the Ag filament (Figure 6(b))
5. Growth of Ag filament to make an electronic contact with AgNW electrode, that is, the cell has switched to the LRS. (Figure 6(c))

The cell maintains its state unless a sufficient voltage of negative bias is applied. A RESET process occurs if an applied negative voltage is greater than the electrochemical dissolution energy of the Ag filament.

6. Electrochemical dissolution of the metal filament, that is, the cell has switched to the initial HRS. (Figure 6(d))

In this paper, we focus on application of transparent electrode for simple fabrication of FT-RRAM. Therefore, for further information of detailed mechanism and microscopic investigation, a number of research group has deeply analyzed the mechanism of cation-based RRAM by a variety method including TEM, AFM, XPS, and so on [5-7].

References

(1) Zhu, B. et al. Silk Fibron for Flexible Electronic Devices. *Adv. Mater.* **28**, 4250-4265 (2016).

(2) Pradhan, S. K. et al. Resistive Switching Behavior of Reduced Graphene Oxide Memory Cells for Low Power Nonvolatile Device Application. *Sci. Rep.* **6**, 26763 (2016).

(3) Jang, B. C. et al. Flexible Nonvolatile Polymer Memory Array on Plastic Substrate via Initiated Chemical Vapor Deposition. *ACS Mater. Interfaces* **8**, 12951-12958 (2016).

(4) Gu, C. & Lee, J. -S. Flexible Hybrid Organic-Inorganic Perovskite Memory. *ACS Nano* **10**, 5413-5418 (2016).

(5) Waser, R., Dittmann, R., Staikov, G. & Szot, K. Redox-based Resistive Switching Memories – Nanoionic Mechanisms, Prospects, and Challenges. Adv. Mater. **21**, 2632-2663 (2009).

(6) Valov, I., Waser, R., Jameson, J. R. & Kozicki M. N. Electrochemical Metallization Memories-Fundamentals, Appliations, Prospects. Nanotecnology **22**, 254003 (2011).

(7) Lu, W., Jeong, D. S., Kozicki, M. & Waser, R. Electrochemical Metallization Cell-Blending Nanoionics into Nanoelectronics? MRS Bull. **37**, 124-130 (2012).
